# A dataset of CMIP6-based climate scenarios for climate change impact assessment in Great Britain

**DOI:** 10.1016/j.dib.2024.110709

**Published:** 2024-07-04

**Authors:** Mikhail A. Semenov, Nimai Senapati, Kevin Coleman, Adrian L. Collins

**Affiliations:** a Sustainable Soils and Crops, Rothamsted Research, West Common, Harpenden AL5 2JQ, United Kingdom; b Net Zero and Resilient Farming, Rothamsted Research, West Common, Harpenden AL5 2JQ, United Kingdom; c Net Zero and Resilient Farming, Rothamsted Research, North Wyke, Okehampton, Devon EX20 2SB, United Kingdom

**Keywords:** Climate scenarios, Downscaling, LARS-WG, CMIP6, SSPs

## Abstract

Climate change is a critical issue in the 21st century. Assessment of the impacts of climate change is beneficial for assisting advanced recommendations for adaptations. Climate change impact assessments require high quality local-scale climate scenarios. The future climate projections from Global Climate Models (GCMs) are problematic to use at local scale due to their coarse spatial and temporal resolution, and existing biases. It is important to have climate change scenarios based on the GCMs ensemble downscaled to local scale to account for inherent uncertainty in climate projections, and to have a sufficient large number of years to account for inter-annual climate variability and low frequency, but high impact, extreme climatic events. A dataset of future climate change scenarios was therefore generated at 26 representative sites across Great Britain based on the latest CMIP6 multi-model ensemble downscaled to local-scale by using a stochastic weather generator, LARS-WG 8.0. The data set consists of climate scenarios of daily weather of 1,000 realizations of typical years for a baseline, and very near (2030) and near-future (2050) climates, based on five GCMs and two emission scenarios (Shared Socioeconomic Pathways - SSPs *viz*. SSP2-4.5 and SSP5-8.5). A total of 15 GCMs from the CMIP6 ensemble were integrated in LARS-WG 8.0. LARS-WG downscales future climate projections from the GCMs and incorporates changes at local scale in the mean climate, climatic variability, and extreme events by modifying the statistical distributions of the weather variables at each site. Based on the performance of the GCMs over northern Europe and their climate sensitivity, a subset of five GCMs was selected, *viz*.; ACCESS-ESM1-5, CNRM-CM6-1, HadGEM3-GC31-LL, MPI-ESM1-2-LR and MRI-ESM2-0. The selected GCMs are evenly distributed among the full set of 15 GCMs. The use of a subset of GCMs substantially reduces computational time, while allowing assessment of uncertainties in impact studies related to uncertain future climate projections arising from GCMs. The 1000 years of daily weather for the baseline, as well as for very near and near-future climate change scenarios, are essential for estimating inter-annual variation, and for detecting low frequency, but high impact, extreme climatic events, such as heat waves, floods and droughts. The present dataset can be used as an input to climate change impact models in various fields, including, land and water resources, agriculture and food production, ecology and epidemiology, and human health and welfare. Researchers, breeders, farm managers, social and public sector leaders, and policymakers may benefit from this new dataset when undertaking impact assessments of climate change and decision support for mitigation and adaptation to climate change.

Specifications Table

**Table:** 

Subject	Climatology; Earth and Planetary Sciences;
Specific subject area	Climate change; extreme climatic events, downscaling of climate data from global climate models and generating daily climate scenarios at local scale.
Type of data	Table (.dat file and .st file) Raw model generated data (txt files)
Data collection	**CMIP6 ensemble data:** Future climate projections from the CMIP6 ensemble were obtained from the Copernicus Climate Data Store https://cds.climate.copernicus.eu/cdsapp#!/dataset/projections-cmip6?tab=form **Observed historical weather:** Observed historical daily weather data at 26 representative sites across the UK (see Table 1 and Fig. 1) was obtained from the UK Met Office [1]. **Baseline and future climate scenarios:** One thousand yearly realisations of daily weather for baseline, and very near and near-future climate scenarios at 26 selected sites were generated by using a stochastic weather generator (LARS-WG 8.0), based on observed data and climate projections from the CMIP6 ensemble.
Data source location	Primary data sources: (i) Observed or historical climate data: UK Met Office [1]. (ii) Future climate projections: CMIP6 ensemble (see Table 2), Copernicus Climate Data Store https://cds.climate.copernicus.eu/cdsapp#!/dataset/projections-cmip6?tab=form
Data accessibility	The data [2] is available from – Repository name: Zenodo Data identification number: https://doi.org/10.5281/zenodo.10556986 Direct URL to data: https://zenodo.org/records/10556986

## 1 Value of the Data

• The dataset [2] provides daily climate scenarios of 1000 realizations of typical years for a baseline, and CMIP6-based local-scale climate scenarios for the very near (2030) and near-future (2050) at 26 representative sites across Great Britain (GB).
• A large number of realisations of daily climate data (1000 years) for a baseline, as well as very near and near-future scenarios, are essential to estimate inter-annual variation and to detect low frequency, but high impact, extreme climatic events, such as heat waves, floods and droughts.
• Future climate scenarios from multiple GCMs are required to estimate uncertainty propagation in future impact assessments from the uncertainty inherent in climate projections due to differences among GCMs.
• Researchers, breeders, farm managers, social and public sector leaders, and policymakers may benefit from this new dataset for impact assessment and decision support associated with mitigation and adaptation strategies [3].
• The new data can be used as an input to climate change impact models used in various fields, including land and water resources, agriculture and food production, ecology and epidemiology, and human health and welfare.

## 2 Background

Existing climate change, including increasing air temperatures, changing precipitation patterns, and rising frequency and intensity of extreme climatic events such as extreme heat waves, droughts and flooding, is a critical issue in the 21st century [4]. Climate change impact studies are essential for assessing the severity of future climate change impacts and for supporting recommendations for mitigation strategies in advance [5,6]. Future climate change scenarios are therefore crucial for different impact studies. The coarse spatial and temporal resolutions of Global Climate Models (GCMs) outputs make it problematic to use them at local scale, and the performances of GCMs also vary across spatial scales globally [7]. The uncertainty and inherent unpredictability of changing weather patterns make climate change assessment challenging. There is substantial uncertainty in future climate projections among GCMs, such as in the latest Coupled Model Intercomparison Project Phase 6 (CMIP6) ensemble [8]. It is important to have a minimum workable ensemble of GCMs, covering the full distribution and climate sensitivity, downscaled to local scale for climate change impact studies. It is also important to have climate scenarios based on a sufficiently large number of years to account for inter-annual climate variability and extreme climatic events. The present dataset of climate scenarios [2] was generated at local-scale over GB to facilitate climate change impact assessments by providing 1000 yearly realizations of daily weather for the baseline, and very near and near-future climate scenarios, based on five GCMs and two emission scenarios.

## 3 Data Description

This dataset [2] provides CMIP6-based local-scale climate scenarios for climate change impact assessments for GB. The dataset consists of daily weather of 1000 realizations of typical years for a baseline and future climate change scenarios at 26 representative sites across GB. The baseline climate at each site was generated by using a stochastic weather generator (LARS-WG 8.0) [7,9,10], based on observed daily weather (1985–2015). The future climate scenarios were generated at each site by using LARS-WG 8.0, based on climate projections from five selected GCMs from the CMIP6 ensemble [8], two Shared Socioeconomic Pathways (SSPs) (SSP2-4.5 and SSP5-8.5) [11,12] and two-time periods, viz. very near-future (2030) and near-future (2050).

**Fig. 1:**
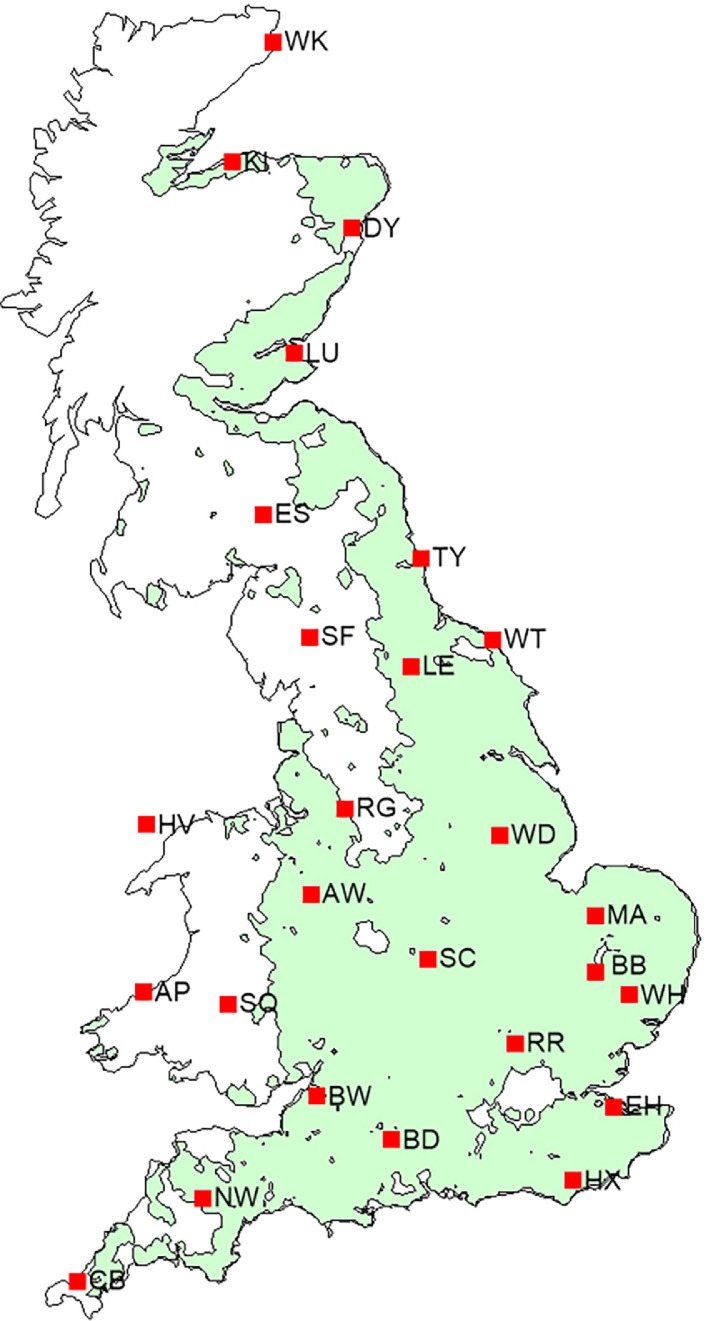
The selected representative sites of the climate dataset [2] across Great Britain. The green shading represents arable land.

The dataset [2] consists of 26 zipped folders for 26 representative sites across GB, with the file name starting with the site abbreviation (Table 1 and Fig. 1) followed by “5GCM1000”, based on the future projections from the five GCMs and two SSPs emission scenarios. Each site folder includes one baseline climate file (.dat), 20-future climate scenarios (5-GCMs × 2-SSPs × 2-periods) files (.dat), and 21 site meta-files (.st) containing information about a site and a generated scenario. The baseline climate file (.dat) name consists of the site abbreviation and the abbreviation “WG” meaning generated by LARS-WG. The baseline climate contains daily weather for 1000 years based on observed weather for 31-years spanning 1985–2015. The future climate scenarios file (.dat) name begins with the site abbreviation, followed by the name of the GCM (Table 2), site abbreviation, SSP, time-period, and abbreviation WG. Two SSPs, viz. SSP2-4.5 and SSP5-8.5, are named as ssp245 and ssp585, whereas the two-time periods 2030 and 2050 are named as 2021–2040 and 2041–2060 (see Methods). Each combination of future climate scenario (GCM × SSP × period) contains daily weather for 1000 years. The files with the .st extension have the same name as the baseline and future climate scenario files (.dat), contain site information (latitude, longitude and altitude), atmospheric CO_2_ concentration, climate file names, and the format of data in the climate file (i.e., name and sequence of climatic variables). The full names and units of climate variables can be found in Table 3.

**Table 1:** The selected representative sites across Great Britain.

Number	Site	Site abbreviation	Latitude (°)	Longitude (°)	Altitude (m)
1	Aberporth	AP	52.14	−4.57	133
2	Shawbury	AW	52.79	−2.66	72
3	Brooms Barn	BB	52.27	0.57	75
4	Boscombe Down	BD	51.16	−1.75	126
5	Bristol	BW	51.45	−2.60	42
6	Camborne	CB	50.22	−5.33	87
7	Dyce	DY	57.21	−2.20	65
8	East Hamsted	EH	51.38	0.78	75
9	Eskdalemuir	ES	55.31	−3.21	242
10	Holyhead Valley	HV	53.25	−4.54	10
11	Herstmonceux	HX	50.89	0.32	52
12	Kinloss	KI	57.65	−3.56	5
13	Leeming	LE	54.30	−1.53	32
14	Leuchars	LU	56.38	−2.86	10
15	Marham	MA	52.65	0.57	21
16	North Wyke	NW	50.77	−3.90	177
17	Ringway	RG	53.36	−2.28	33
18	Rothamsted	RR	51.80	−0.35	128
19	Church Lawford	SC	52.36	−1.33	107
20	Shap Fell	SF	54.50	−2.68	255
21	Sennybridge	SQ	52.06	−3.61	307
22	Tynemouth	TY	55.02	−1.42	33
23	Waddington	WD	53.18	−0.52	68
24	Wattisham	WH	52.12	0.96	89
25	Wick	WK	58.45	−3.09	36
26	Whitby	WT	54.48	−0.60	41

**Table 2:** The five Global Climate Models (GCMs) from the Coupled Model Intercomparison Project Phase 6 (CMIP6) multi-model ensemble used in the future climate change scenarios for Great Britain.

GCM	Research centre	Country	Grid resolution: latitude × longitude	References
ACCESS-ESM1-5	Commonwealth Scientific and Industrial Research Organisation (CSIRO)	Australia	1.25° × 1.875°	[13]
CNRM-CM6-1	Centre National de Recherches Meteorologiques (CNRM), Centre Europeen de Recherche et de Formation Avancee en Calcul Scientifique (CERFACS)	France	1.40° × 1.406°	[14]
HadGEM3-GC31-LL	UK Met Office Hadley Centre (MOHC)	UK	1.25° × 1.88°	[15]
MPI-ESM1-2-LR	Max Planck Institute for Meteorology (MPI-M)	Germany	1.39° × 1.41°	[16]
MRI-ESM2-0	Meteorological Research Institute (MRI)	Japan	1.113° × 1.125°	[17]

**Table 3:** The variables, abbreviations and units as used in the dataset.

Variable	Julian day/ Day of year	Minimum temperature	Maximum temperature	Rainfall	Solar radiation
abbreviation	JDAY	MIN	MAX	RAIN	RAD
Unit	N /A	° C	°C	mm day^−1^	MJ m^−2^ day^−1^

## 4 Experimental Design, Materials and Methods

*Representative sites across the UK*. A total of 26 representative sites across GB were selected for this dataset [2] from an available 85 climate stations within the Met Office network [1], including only sites which reported less than 10 % missing values for temperature and precipitation, providing broad and even coverage across GB to cover almost all arable land (Table 1 and Fig. 1).

**Fig. 2:**
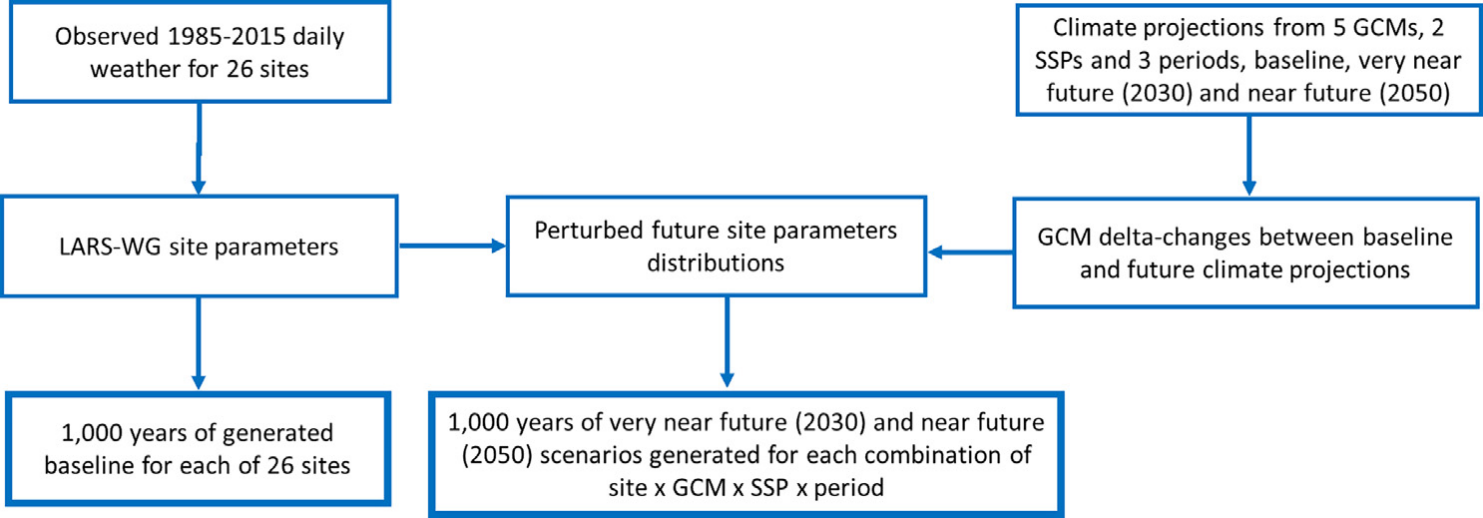
A flow chart diagram for generation of baseline and future climate scenarios based on 5 GCMs from CMIP6 and 2 SSPs emission scenarios.

*Baseline climatic scenarios.* The 31 years (1985–2015) of baseline daily observed weather data were available at each selected site [1]. To generate a baseline, observed weather data were used to estimate site-specific climatic parameters needed for the LARS-WG 8.0 stochastic weather generator [7,9,10]. To account for inter-annual climatic variability and to detect low frequency, but high impact, extreme climatic events, such as heat waves, droughts and floods, 1000 years of daily weather at each site were generated using LARS-WG based on estimated site parameters; hereafter, defined as the ‘baseline climate’ (Fig. 2). The baseline climate has statistical characteristics similar to the observed weather at each site, with probability distributions close to those of the observed climate. An accurate reproduction of climatic variability using LARS-WG has been demonstrated in previous studies [7,18].

**Fig. 3:**
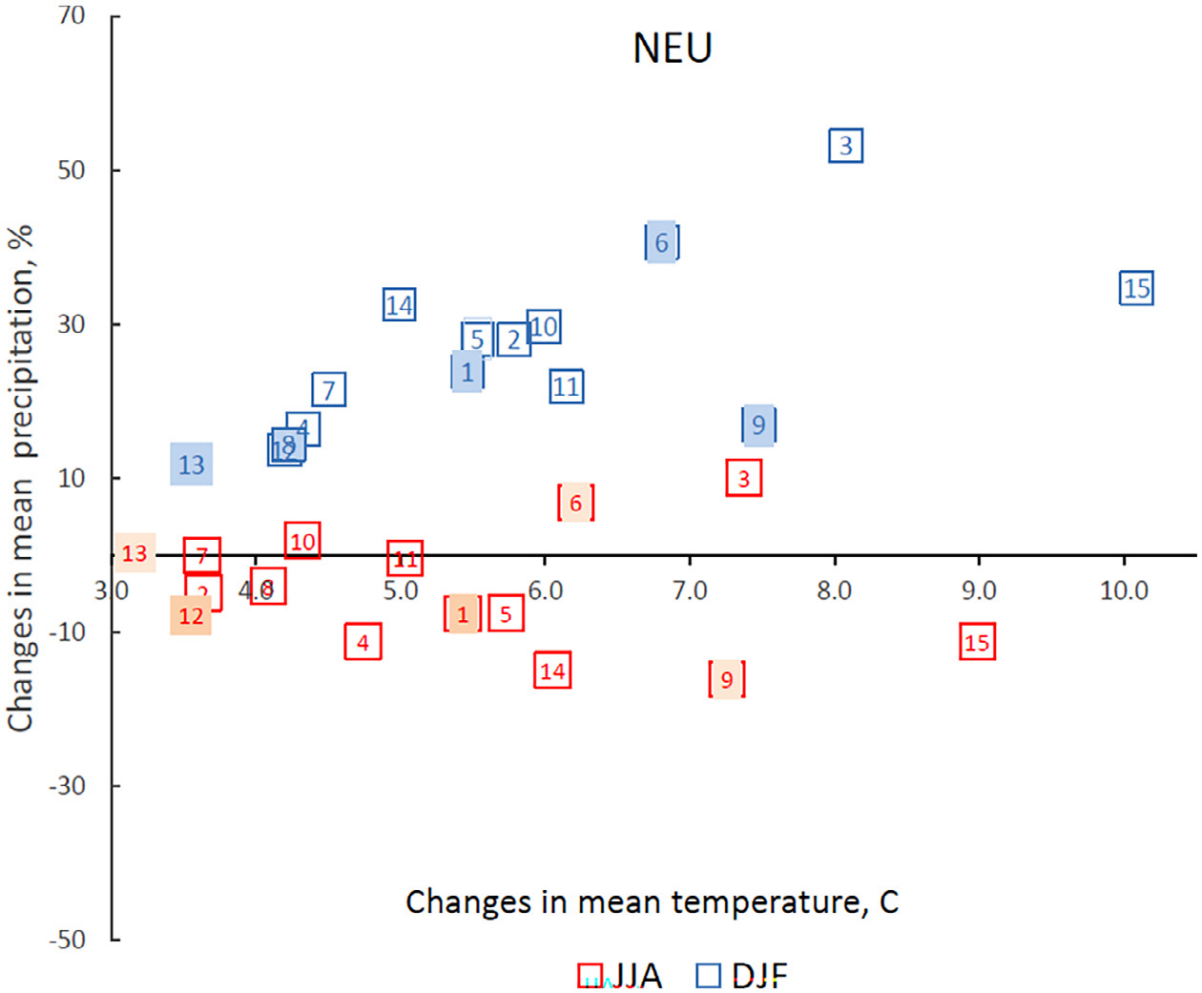
Climate sensitivity of 15 global climate models (GCMs) from the CMIP6 ensemble over Northen Europe (NUE). Absolute changes in annual mean temperatures, °C, and relative changes in precipitation, %, for 2081–2100 (SSP5-8.5) compared with baseline for 15 GCMs from CMIP6 integrated in LARS-WG 8.0 calculated for NEU for winter (DJF) and summer (JJA). Five highlighted GCMs were selected for scenario construction for impact assessment in GB representing the uncertainty in CMIP6 climate projections: ACCESS-ESM1-5 (1), CNRM-CM6-1 (6), HadGEM3-GC31-LL (9), MPI-ESM1-2-LR (12) and MRI-ESM2-0 (13).

*Future climate scenarios based on the CMIP6 ensemble.* Future climate projections were based on GCMs from the CMIP6 ensemble [8]. A total of 15 GCMs from the CMIP6 ensemble were integrated into LARS-WG 8.0. Based on performance over northern Europe including the UK, climate sensitivity and the distribution of GCMs, a subset of five GCMs was selected for the present dataset, *viz*. ACCESS-ESM1-5, CNRM-CM6-1, HadGEM3-GC31-LL, MPI-ESM1-2-LR and MRI-ESM2-0 (Table 2 and Fig. 3) [10,19,20]. The selected five GCMs are evenly distributed among the 15 GCMs, capturing uncertainty in climate projections from the CMIP6 ensemble (Fig. 3). The use of a subset of five GCMs substantially reduces computational time, while allowing assessment of uncertainties in impact studies related to uncertainty in future climate projections due to the GCMs. Two different future climate change scenarios, defined as Shared Socioeconomic Pathways (SSPs), were selected for this dataset to cover the range of possible future development of anthropogenic drivers of climate change, *viz*. (i) an intermediate GHG emission scenario: SSP2-4.5 – Middle of the Road (medium challenges to mitigation and adaptation), with an additional radiative forcing of 4.5 W m^−^² by the year 2100; and (ii) a very high GHG emission scenario: SSP5-8.5 – Fossil-fuelled Development – Taking the Highway (high challenges to mitigation, low challenges to adaptation), with an additional radiative forcing of 8.5 W m^−^² by the year 2100 [11,12]. Two-time periods were used comprising very near-future (2030) and near-future (2050). LARS-WG downscales climate projections from the GCMs and incorporates changes at local scale in the mean climate, climatic variability and extreme events derived from the GCMs by modifying the statistical distributions of the weather variables at each site [7,10]. The monthly output from GCMs during 2021–2040 for very-near future and 2041–2060 for near future were used to calculated delta-changes in climatic variables which were used to perturbed site parameter distributions of LARS-WG for baseline 1985–2015 (Fig. 2). For each site, GCM and SSP, 1000 years of daily weather data were generated using LARS-WG 8.0, based on climate projections from GCMs for the 2021−2040 period, hereafter defined as the ‘2030-climate’ or very near-future climate. Similarly, for each site, GCM and SSP, 1000 years of daily future weather data were generated based on climate projections for the 2041−2060 period, hereafter defined as the ‘2050-climate’ or near-future climate. Therefore, 1000 years for 2021–2040 or 2041–2060 periods means 1000 sample-years typical for these periods. Adequate performance of LARS-WG in terms of capturing climate change, including extreme climatic events, at local scale, has been reported in various previous studies [7,18,21,22].

## Code Availability

The stochastic weather generator LARS-WG 8.0 used to generate this new dataset is available from https://sites.google.com/view/lars-wg/ or https://doi.org/10.5281/zenodo.10632391.

## Limitations

Not applicable.

## Ethics Statement

The authors confirm that the current work does not involve human subjects, animal experiments, or any data collected from social media platforms.

## CRediT authorship contribution statement

**Mikhail A. Semenov:** Conceptualization, Methodology, Software, Validation, Data curation, Writing – review & editing. **Nimai Senapati:** Conceptualization, Writing – original draft, Writing – review & editing. **Kevin Coleman:** Conceptualization, Writing – review & editing. **Adrian L. Collins:** Conceptualization, Writing – review & editing.

## Data Availability

CMIP6-based local-scale climate scenarios for impact assessment in Great Britain. (1.0) (Original data) (Zenodo).

## References

[1] Met Office, The UK climate - Synoptic and climate stations. UK. 2023.

[2] Semenov M.A., Senapati N., Collins A.L. (2024).

[3] Senapati N., Chabbi A., Smith P. (2018). Modelling daily to seasonal carbon fluxes and annual net ecosystem carbon balance of cereal grain-cropland using DailyDayCent: a model data comparison. Agric. Ecosyst. Env..

[4] Senapati N., Halford N.G., Semenov M.A. (2021). Vulnerability of European wheat to extreme heat and drought around flowering under future climate. Environ. Res. Lett..

[5] Putelat T. (2021). Local impacts of climate change on winter wheat in Great Britain. R. Soc. Open Sci..

[6] Senapati N. (2020). Substantial increase in yield predicted by wheat ideotypes for Europe under future climate. Clim. Res..

[7] Semenov M.A. (2010). ELPIS: a dataset of local-scale daily climate scenarios for Europe. Clim. Res..

[8] Eyring V. (2016). Overview of the Coupled Model Intercomparison Project Phase 6 (CMIP6) experimental design and organization. Geosci. Model Dev..

[9] Semenov M.A. (2024).

[10] Semenov M.A., Stratonovitch P. (2015). Adapting wheat ideotypes for climate change: accounting for uncertainties in CMIP5 climate projections. Clim. Res..

[11] Riahi K. (2017). The Shared Socioeconomic Pathways and their energy, land use, and greenhouse gas emissions implications: an overview. Glob. Environ. Change..

[12] O'Neill B.C. (2016). The Scenario Model Intercomparison Project (ScenarioMIP) for CMIP6. Geosci. Model Dev..

[13] Ziehn T. (2019).

[14] Voldoire A. (2018).

[15] Roberts, M. MOHC HadGEM3-GC31-LL model output prepared for CMIP6 HighResMIP. 2017.

[16] Wieners K.-H. (2019).

[17] Yukimoto S. (2019).

[18] Gitau M.W., Mehan S., Guo T. (2018). Weather generator effectiveness in capturing climate extremes. Environ. Process..

[19] Bradshaw C. (2023).

[20] Parding K.M. (2020). GCMeval – An interactive tool for evaluation and selection of climate model ensembles. Clim. Serv..

[21] Semenov M.A. (2008). Simulation of extreme weather events by a stochastic weather generator. Clim. Res..

[22] Semenov M.A., Brooks R.J., Barrow E.M., Richardson C.W. (1998). Comparison of the WGEN and LARS-WG stochastic weather generators for diverse climates. Clim. Res..

